# Childhood Adversities and Unmet Needs of Older Chinese Adults: The Mediation Effects of Family Relationships

**DOI:** 10.1177/01640275211048237

**Published:** 2021-10-13

**Authors:** Bo Hu, Mingyu Wei

**Affiliations:** 1 Care Policy and Evaluation Centre (CPEC), Department of Health Policy, 4905 London School of Economics and Political Science, London, UK; 2 School of Arts and Cultures, 5994 Newcastle University, Newcastle, UK

**Keywords:** unmet needs, childhood adversities, family relationships, mediation analysis, China, older adults

## Abstract

Ensuring equality and adequacy of care for older adults is vitally important. This study investigates the relationships between childhood adversities and unmet long-term care needs of older adults in China and the mediation effects of family relationships. The data came from a nationally representative sample of older Chinese adults aged 60 and over with long-term care needs (*N* = 2186). We conducted mediation analyses and decomposed the total effects of childhood adversities on unmet needs into direct and indirect effects. The probability of unmet needs is significantly higher among older adults experiencing childhood adversities. Satisfaction with marriage mediates the association between childhood adversities and unmet personal care needs. Relationships with children mediate the association between childhood adversities and unmet domestic care needs. The causes of unmet needs can be traced back to early life, which underscores the importance of concerted efforts in family, education and long-term care policies to tackle unmet needs.

## Introduction

Long-term care involves assistance and services that help people perform daily activities such as dressing, eating and cooking and is crucial for older adults who experience a loss of functional capability in later life. Unmet long-term care needs occur when help is needed but is either non-existent or insufficient, not only posing a great threat to personal well-being but also indicating social injustice in modern society (Hu & Li, 2020; Williams et al., 1997).

Due to global population ageing, demand for long-term care is set to rise rapidly worldwide. In the case of China, there are currently 250 million older adults aged 60 and over, accounting for 18% of the total population. However, Chinese society faces a plummeting supply of care from family members as the impact of the one-child policy implemented in the 1980s starts to materialise and the average household size continues to decrease (Zeng et al., 2013). It is projected that the number of working-age adults will decrease by 14% whereas the number of older adults will increase by 74% by 2040 (United Nations, 2019). Ensuring the adequacy of long-term care for older adults is currently at the top of the Chinese government’s agenda (Peng et al., 2015).

In this context, it is vitally important to have a systematic understanding of the factors associated with unmet long-term care needs in the older population. Although a large number of studies have investigated the contemporary predictors of unmet needs, no research has been done to examine the influences of early life experiences. The life-course perspective stresses that ageing is a developmental process that spans a person’s entire life (Elder et al., 2003). In particular, childhood is a critical period of life because massive biological and psychological programming takes place in this phase. Experiences and events in childhood are intrinsically linked with life outcomes in subsequent ones.

The majority of long-term care responsibilities are undertaken by family members. The utilisation of long-term care and unmet care needs are a manifestation of the caring relationships between older adults with care needs and their family caregivers. Experiences in childhood shape children’s interpretation of and expectations from family relationships and affect their ability to fulfil multiple roles in the social networks in adulthood (Mikulincer & Shaver, 2009; Wolke et al., 2013). Deeply embedded in those relationships, people’s support-seeking behaviour and attitude towards social support are transformed by events and memories in the past and take shape throughout the life course, which has profound consequences for care utilisation in old age (Mikulincer & Shaver, 2007, pp.195–199). Ignoring the influences of early life experiences will run the risk of leaving a large number of cases of unmet needs unexplained.

Drawing on data from a Chinese national survey, this study investigates the association between adverse experiences in childhood and unmet long-term care needs in old age. Our attention is focussed on the pathways through which the quality of family relationships mediates this association. The research findings will contribute to the understanding of care utilisation among older adults from the standpoint of relationships with spouse and children and join the ongoing debate about the social policy of ageing from a life course perspective.

## Literature Review

### Determinants of Unmet Long-Term Care Needs

The existing literature makes a conceptual distinction between absolute and relative unmet long-term care needs (Vlachantoni, 2019). The former refers to the situation where an older person has a care need but does not receive any help at all. The latter recognises the fact that people who receive care may not be satisfied with the help or need more help, which enables researchers to explore whether a care need is undermet or fully met (Kennedy, 2001; Peng et al., 2015). A large body of literature has examined the factors associated with unmet long-term care needs. The identification of those factors is often guided by the behavioural model of care utilisation (Andersen & Newman, 2005). According to this model, the determinants of unmet needs can be divided into three groups: predisposing factors, need factors and enabling factors.

Predisposing factors are the individual-level characteristics such as age and gender that affect the propensity of care utilisation prior to the onset of care needs. The relationship between predisposing factors and unmet needs is found to vary across different countries. For example, studies conducted in the US and Canada (Dubuc et al., 2011; Kennedy, 2001) showed that females are more likely than males to have unmet needs, whereas Momtaz et al. (2012) reported that older males in Malaysia have a higher probability of experiencing unmet needs.

An investigation into the determinants of unmet care needs is conditioned on the identification of people with care needs. In many studies, long-term care needs are measured by functional limitations, namely difficulties in performing activities of daily living (ADLs) and instrumental activities of daily living (IADLs) (Williams et al., 1997). ADL functional limitations relate to the personal care needs of older adults (Katz et al., 1970), whereas IADL functional limitations capture domestic care needs and demonstrate the ability of older adults to adapt to the environment (Lawton & Brody, 1969; Spector & Fleishman, 1998).

The relationships between functional limitations and unmet care needs reported in the literature have been inconclusive. Kennedy (2001) and Casado et al. (2011) have found that American older adults with more severe functional limitations are more likely to have unmet needs. In contrast, Liu et al. (2012) reported that more severe functional limitations are associated with a lower probability of experiencing unmet needs in Taiwan.

Enabling factors are the resources that older adults possess or mobilise to access long-term care. They can be further divided into socioeconomic status and social support networks. Several studies (Kröger et al., 2019; Peng et al., 2015; Wilkinson-Meyers et al., 2014) have shown that older adults with a lower income are more likely to have unmet needs. In high-income countries, professional/formal caregivers and family caregivers join forces to meet care needs of older adults. Recipients of formal long-term care often have to pay towards the care costs, so care affordability poses a major risk for unmet needs. In this case, government support for older adults with lower financial means plays an important role in improving the affordability of services, which helps to alleviate unmet needs in the older population. Kemper et al. (2008) investigated the impacts of Medicaid home care spending in the US and found that more generous financial support from the government reduces the proportion of older adults with unmet personal care needs.

There is a consensus in the literature that the availability of social networks, especially family caregivers, has a large impact on care utilisation (Williams et al., 1997). Family members, in particular spouse and adult children, are usually the primary caregivers of older adults, so unmet needs are strongly associated with living arrangements and marital status. Almost all of the studies discussed above have found that older adults living alone are significantly more likely than those living with relatives to experience unmet needs. Among those living with other people, widowed or separated older adults are more likely than married older adults to have unmet needs (Lima & Allen, 2001; Vlachantoni, 2019), which points to the indispensable role of spouse care in the care networks of older adults.

In addition to the availability of family caregivers, the quality of relationships with family members also matters. Van Groenou and De Boer (2016) proposed the Informal Care Model and theorised that family members’ decision to provide care depends heavily upon their personal bond with the people with care needs: the stronger the bond, the greater the likelihood of providing care. Silverstein et al. (1995) found that adult daughters who feel close to their older parents and have good communication with them are more likely to provide care.

The Behavioural Model of Care Utilisation and the Informal Care Model are useful theoretical frameworks that help researchers understand the characteristics of older adults living with unmet care needs. While both have placed great emphasis on the contemporary factors influencing the caring relationships between care recipients and caregivers, little attention has been paid to the historical contexts, events and experiences that preconditioned those relationships. None of the empirical studies reviewed above has considered early life experiences as a risk factor of unmet needs either. There is a research gap in the existing literature.

### Childhood Adversities and Later Life Outcomes

A growing body of literature has demonstrated that childhood adversities are associated with poor health and a variety of health conditions in later life, which could lead to the onset and progression of long-term care needs (Hughes et al., 2017). Schafer and Ferraro (2012) found that people experiencing a higher number of childhood adversities are less likely to maintain a disease-free status in old age. Hempel et al. (2021) reported that low socioeconomic status and poor health in childhood are strongly associated with poor physical and mental health in old age. An analysis conducted by Hu, 2021 showed that experiencing multiple childhood adversities significantly reduce the likelihood of achieving healthy ageing. Older adults reporting multiple adversities in childhood are significantly more likely to have functional limitations or long-term care needs.

Childhood adversities also have adverse consequences for key social outcomes such as social functioning and social relationships. Parents are the primary caregivers of babies and children. The presence and quality of parental care are crucial to the development of child attachment (Bowlby, 1997). A secure attachment means that children are comfortable with intimacy, dependency and reciprocity in relationships, which will have long-lasting effects on forming and maintaining a supportive social network in the future. Childhood adversities can take many forms, but most of them either directly relate to or act as the context of a lack of parental care. Growing up with parents who are abusive or show little affection are adversities of the former category, whereas material deprivation and parental mental health are examples of the latter (Stansfeld et al., 2008). In both cases, childhood adversities disrupt the development of attachment, which leads to either over-responsiveness or under-responsiveness to social relationships. Some may constantly feel anxious about the stability of their social relationships, while others may avoid or be dismissive of social relationships (Mikulincer & Shaver, 2009). Insecure attachment leads to difficulties in maintaining a good social relationship with family members and friends in later life.

Drawing on the attachment theory, an increasing number of empirical studies have shown that childhood adversities are associated with poor family relationships in adulthood. For example, Rodgers (1996) studied a sample of 3262 British adults aged between 36 and 43 and found that those with childhood experiences of low care and high control from parents were more likely to be divorced or separated and less likely to rate their social networks as good. These findings were largely supported in later studies focussing on people from other countries or specific demographic groups. Umberson et al. (2005) found that marital quality was more volatile for people growing up with high levels of family stress. Brown et al.’s (2008) work showed that women with neglective childhood experiences prior to the age of 17 were more likely to have interpersonal difficulties decades later. Ford et al. (2011) found that people experiencing childhood adversities such as parental divorce, physical abuse and sexual abuse were more likely to have a smaller social network size and report negative aspects of family relationships including worries, problems and stress in midlife. Umberson et al. (2014) reported that childhood adversities were associated with more strained relationships with family and friends and less support from them in the US.

### Hypotheses of the Present Study

The existing literature on attachment security and family relationships suggests that people experiencing multiple adversities in childhood often struggle to maintain a good relationship with family members. The tendency to run into problematic relationships are difficult to reverse in later life because insecure attachment, the underlying driver of poor relationships, takes root in childhood and becomes relatively stable in adulthood (Crowell et al., 2002). It seems that the negative impacts of childhood adversities on family relationships can follow people throughout the life course and last until old age.

Meanwhile, previous studies on the determinants of unmet needs have underscored the central role of family relationships in shaping the decisions of care provision and care use. Poor family relationships indicate a decline in the quality of social networks and constitute a salient risk factor for unmet needs in the older population. Combining the two bodies of literature, we propose two hypotheses to be tested in the present study:

(1) Older adults experiencing more adversities in childhood are more likely to have unmet long-term care needs.
(2) The association between childhood adversities and unmet needs is mediated by poor relationships of older adults with key family members including spouse and children.

## Methods

### Data and Sample

The data for this study came from the China Health and Retirement Survey (CHARLS), which collected ageing and health-related information from a nationally representative sample of Chinese people aged 45 and over. The baseline survey was conducted in 2011 and included 17,500 individuals in 150 counties across the country. Follow-up studies were conducted in 2013 and 2015, respectively. A life history survey was conducted in 2014, which focussed on people’s childhood and midlife experiences and events.

A total of 9208 older adults aged 60 and over participated in both the life history survey and the 2015 follow-up survey. We conducted a preliminary analysis and found that the age and gender structure of this sample is highly consistent with that reported in the official statistics (United Nations, 2019), indicating that the sample is representative of the older Chinese population. We then excluded 7022 people who did not have long-term care needs. The focus of this study was the 2186 people with care needs (see definitions below).

### Key Measurements

The key variables of interest are unmet long-term care needs, childhood adversities and quality of family relationships. Long-term care needs were measured by functional capability. The CHARLS asked whether respondents could perform five activities of daily living (ADLs; dressing, bathing, eating, transferring and toileting) and five instrumental activities of daily living (IADLs; doing housework, cooking, taking medication, shopping and managing money). For each task, respondents were presented with four options: ‘I have no difficulty in doing it,’ ‘I have difficulty but still can do it,’ ‘I need help’ and ‘I cannot do it.’ To ensure that these tasks were relevant to long-term care needs, the questionnaire emphasised that the difficulties should be attributable to physical, mental, emotional or memory problems and expected to last more than 3 months. The latter two options, namely ‘I need help’ or ‘I cannot do it,’ indicated a functional limitation or a long-term care need for the task. People with at least one ADL functional limitation were considered as having ADL care needs or personal care needs. People with at least one IADL functional limitation were considered as having IADL care needs or domestic care needs. In this study, people with long-term care needs include those with ADL or IADL care needs.

Respondents were then asked whether they received help with each task. No receipt of help in the presence of a care need was defined as having an unmet need with respect to this task. We counted the number of unmet ADL care needs and unmet IADL care needs, respectively. Because only a small number of people had more than two unmet needs, we created two binary unmet needs variables relating to ADL and IADL care, respectively (0 = no unmet needs and 1 = with unmet needs). It can be noted that our measure captures absolute unmet needs (see the *Literature Review* section).

We investigated the family information module of the life history questionnaire and identified 16 types of childhood adversities: (1) parental death, (2) divorce, (3) a parent often feeling anxious, (4) a parent often feeling depressed, (5) severe disability of a parent, (6) a parent suffering from mental illness, (7) lack of affection, (8) parental neglect, (9) physical abuse, (10) parental alcohol misuse, (11) parental drug misuse, (12) parental involvement in criminal activities, (13) frequent domestic violence, (14) being poorer than other families, (15) poor childhood health and (16) frequent bullying victimisation. We coded each adversity in a dichotomous form: 0 = no and 1 = yes. Following Schafer and Ferraro (2012), we counted the number of childhood adversities reported by each survey participant.

The CHARLS 2015 has two questions relating to the quality of family relationships. Married older adults were asked to rate their satisfaction with their relationship with their spouse, and older adults who had living children were asked to rate their satisfaction with their relationships with their children. Both types of relationships were rated on a five-point Likert scale: 1 = completely satisfied, 2 = very satisfied, 3 = somewhat satisfied, 4 = not very satisfied and 5 = not at all satisfied. We created two ordinal variables.

### Control Variables

We controlled for the predisposing, need and enabling factors proposed in the behavioural model of care utilisation. This was done in order to understand whether the influence of childhood adversities was above and beyond key covariates reported in the existing literature. In addition, the extent to which childhood adversities affected unmet needs independent of those covariates will have important policy implications. We included three predisposing factors in the analyses: age, gender and rural-urban residence. The rural-urban residence variable was dichotomised (0 = urban China and 1 = rural China).

We controlled for two need variables: functional limitations and chronic illnesses. Kingston et al. (2012) reported that loss of ability to perform IADL tasks precedes loss of ability to perform ADL tasks. Following this hierarchy, we created an ordinal variable to measure older adults’ long-term care needs. The variable had three levels: IADL care needs only, one ADL care need, and two or more ADL care needs. The CHARLS collected information about 14 types of chronic diseases including hypertension, dyslipidaemia, diabetes, cancer, lung diseases, liver diseases, coronary heart disease, stroke, kidney diseases, stomach diseases, psychiatric problems, memory-related problems, arthritis and asthma. We added up the number of diseases reported by respondents and created a count variable.

The enabling factors consist of the availability of social support and socioeconomic factors. The availability of social support was measured by marital status, living arrangements and the number of living children. The marital status variable was dichotomised: 0 = never married, widowed, separated or divorced and 1 = married. The living arrangements variable had two categories: 0 = living alone and 1 = living with other people in the same household. The number of children is a count variable. We investigated two socioeconomic variables. Older adults were asked to report their highest level of education. They were coded as 0 if they did not receive any formal education and 1 otherwise. Older adults were also asked whether they received a public or private pension. We created a dichotomised variable: 0 = no and 1 = yes.

### Statistical Analysis

We built binary logistic regression models to test the first hypothesis:

(1) $$\ln\left[\frac{\text{Pr}\left(y_{i}=1\right)}{\text{Pr}\left(y_{i}=0\right)}\right]=\alpha +\beta_{1}\times x_{i}+\overset{K}{\underset{k=1}{\sum }}\left(\gamma_{k}\times z_{ki}\right)\;,$$

where $\text{Pr}\left(y_{i}=1\right)$ denotes the probability of having unmet care needs for an individual i, $x_{i}$ is the number of childhood adversities reported by this person and $z_{ki}$ denotes the kth control variable. $\beta_{1}$ and $\gamma_{k}$ are the coefficients for the childhood adversities and control variables, respectively. The odds ratio of unmet needs is denoted by $\frac{\text{Pr}\left(y_{i}=1\right)}{\text{Pr}\left(y_{i}=0\right)}$ , which can be calculated by exponentiating the coefficients. Equation (1) was specified to investigate the total effects of childhood adversities on unmet needs, so mediators were not included in this model. We examined unmet ADL care needs and unmet IADL care needs separately in regression analyses.

To test the second hypothesis, we focussed on married older adults and older adults with living children, respectively. In each case, we modelled the mediating pathways as follows:

(2) $$\;\;\ln\left[\frac{\text{Pr}\left(m_{i}\leq j\right)}{\text{Pr}\left(m_{i}>j\right)}\right]=\tau_{j}+\beta_{2}\times x_{i}+\overset{K}{\underset{k=1}{\sum }}\left(\gamma_{k}\times z_{ki}\right),\;\;\;\;for\;j=1-4\;,$$

(3) $$\ln\left[\frac{\text{Pr}\left(y_{i}=1\right)}{\text{Pr}\left(y_{i}=0\right)}\right]=\alpha +\beta_{3}\times x_{i}+\beta_{4}\times m_{i}+\overset{K}{\underset{k=1}{\sum }}\left(\gamma_{k}\times z_{ki}\right).$$

Equation (2) is an ordinal logistic regression model, whereas equation (3) is a binary logistic regression model. $m_{i}$ denotes the quality of family relationships, the mediating factor between adversities in childhood and unmet needs in older age. $y_{i}$ , $x_{i}$ and $z_{ki}$ have the same denotations as in equation (1). Like in equation (1), we examined unmet ADL care needs and unmet IADL care needs separately.

The existence of mediation effects was indicated by the statistical significance of the indirect effects of childhood adversities on unmet care needs through the quality of family relationships. Equations (2) and (3) are non-linear models, so the coefficients are on different scales and cannot be readily combined together to calculate the indirect effects. We took three approaches to address this issue. First, following the method proposed by Iacobucci (2012), we calculated the z-values of $\beta_{2}$ and $\beta_{4}$ which are measured on the same scale:

$$z\left(\beta_{2}\right)=\;\frac{\beta_{2}}{Se\left(\beta_{2}\right)},z\left(\beta_{4}\right)=\;\frac{\beta_{4}}{Se\left(\beta_{4}\right)}.$$

$Se\left(\beta_{2}\right)$ and $Se\left(\beta_{4}\right)$ are the standard errors of $\beta_{2}$ and $\beta_{4}$ , respectively. Then the z-value of the indirect effect, which has a standard normal distribution (Iacobucci, 2012), was calculated as follows:

$$z_{mediation}=\;\frac{z\left(\beta_{2}\right)\times z\left(\beta_{4}\right)}{\sqrt{z^{2}\left(\beta_{2}\right)+\;z^{2}\left(\beta_{4}\right)+1}}.$$

Second, we adopted the Karlson–Holm–Breen (KHB) method (Karlson et al., 2012). This method involves regressing the mediator (i.e. family relationships) on the childhood adversities and adding the residuals calculated from this regression to equation (1). The KHB method leads to a decomposition of the total effects of childhood adversities into direct effects and indirect effects. We also calculated the percentage of indirect effects out of the total effects, which is an alternative indicator of the importance of the mediation effects.

Third, we re-estimated the coefficients of unmet needs and family relationships using linear probabilities models where the coefficients are by design measured on the same scale. This method also leads to a decomposition of the total effects into direct effects and indirect effects. We calculated the standard errors of the indirect effects through bootstrapping (re-sampling of 1000 times), which enabled us to make statistical inferences about the indirect effects.

The proportion of missing values is negligible for most variables in the dataset. However, 16% of married older adults and 20% of people with children did not provide information on the quality of their family relationships. We evaluated two alternative approaches to address the missingness: (1) using multiple imputation to impute the missing values in these two variables (20 imputed datasets), and (2) excluding the observations with missing values from the analysis (i.e. complete cases analysis). The two approaches led to very similar regression results. We report the imputation-based regression results in the next section. The results based on complete case analysis are reported in the Supplementary appendix.

### Research Findings

Among 2186 people with long-term care needs, 895 people (41%) had ADL care needs (Table 1), 38% of whom (*n* = 344) reported that their care needs were unmet. The average number of childhood adversities was 1.89. There were great variations in their childhood experience: 20% had no childhood adversities, whereas 7% had five or more childhood adversities. Married older adults on average scored 2.60 in terms of their relationship with their spouse. Half reported that they were completely satisfied or very satisfied with their marriage, while 13% reported that they were not very satisfied or not at all satisfied with their marriage. Older adults with living children on average scored 2.44 in terms of their relationships with their children. Fifty-six per cent were completely or very satisfied with the relationships, whereas 10% were not very satisfied or not at all satisfied with the relationships. Fifty-eight per cent of older adults with ADL care needs were aged 70 and over. Seventy-five per cent were living in rural areas. Sixty-nine per cent were married. Eighty-seven per cent were living with other people in the same household. They on average reported 2.4 chronic diseases.

**Table 1.** Sample Characteristics (*N* = 2186).

Variables	People with ADL care needs (*n* = 895)	People with IADL care needs (*n* = 1972)
	Proportions (frequency) or means (frequency)	
Unmet needs		
No	62% (551)	73% (1430)
Yes	38% (344)	27% (542)
Number of childhood adversities	1.9 (854)	1.9 (1891)
Relationships with spouse (range:1–5)	2.6 (482)	2.6 (1128)
Relationships with children (range:1–5)	2.4 (654)	2.5 (1518)
Age		
60–69 years old	41% (369)	45% (881)
70–79 years old	37% (332)	36% (708)
80+ years old	21% (191)	19% (372)
Gender		
Men	41% (366)	39% (774)
Women	59% (529)	61% (1198)
Residence		
Urban areas	25% (221)	21% (419)
Rural areas	75% (667)	79% (1540)
Marital status		
Single	31% (275)	30% (599)
Married	69% (618)	70% (1371)
Number of children	3.8 (895)	3.7 (1972)
Living arrangements		
Living alone	13% (112)	12% (237)
Living with other people	87% (782)	88% (1735)
Care needs		
IADL limitations only	0% (0)	65% (1291)
1 ADL limitation	59% (526)	17% (327)
2+ ADL limitations	41% (369)	18% (354)
Number of chronic diseases	2.4 (895)	2.1 (1972)
Receipt of formal education		
No	43% (382)	46% (909)
Yes	57% (512)	54% (1060)
Receiving pension		
No	28% (255)	29% (563)
Yes	72% (640)	71% (1409)

One thousand nine hundred seventy two out of 2186 people (90%) had IADL care needs, among whom 27% (*n* = 542) had unmet care needs (Table 1). The average number of childhood adversities was 1.94. The overall scores for relationships with spouse and children were 2.61 and 2.46, respectively. People with IADL care needs had similar demographic and socioeconomic characteristics to those with ADL care needs (Table 1). They on average had 2.1 chronic diseases.

Table 2 shows the pairwise Spearman correlation between the key variables. Most of the correlations were statistically significant. In particular, childhood adversities were strongly correlated with both unmet needs and family relationships. The exceptions were the correlation between the relationship with spouse and unmet IADL care needs (*P*-value = .60) and the correlation between relationships with children and unmet ADL care needs (*P*-value = .21).

**Table 2.** Pairwise Spearman Correlation Between Key Variables of Interest.

	1	2	3	4	5
1. Unmet ADL care needs	—				
2. Unmet IADL care needs	0.34***	—			
3. Childhood adversities	0.11***	0.08***	—		
4. Relationships with spouse	0.16***	0.02	0.12***	—	
5. Relationships with children	0.05	0.07**	0.11***	0.43***	—

Notes: *P < .05, **P < .01, ***P < .001.

**Figure 1.**
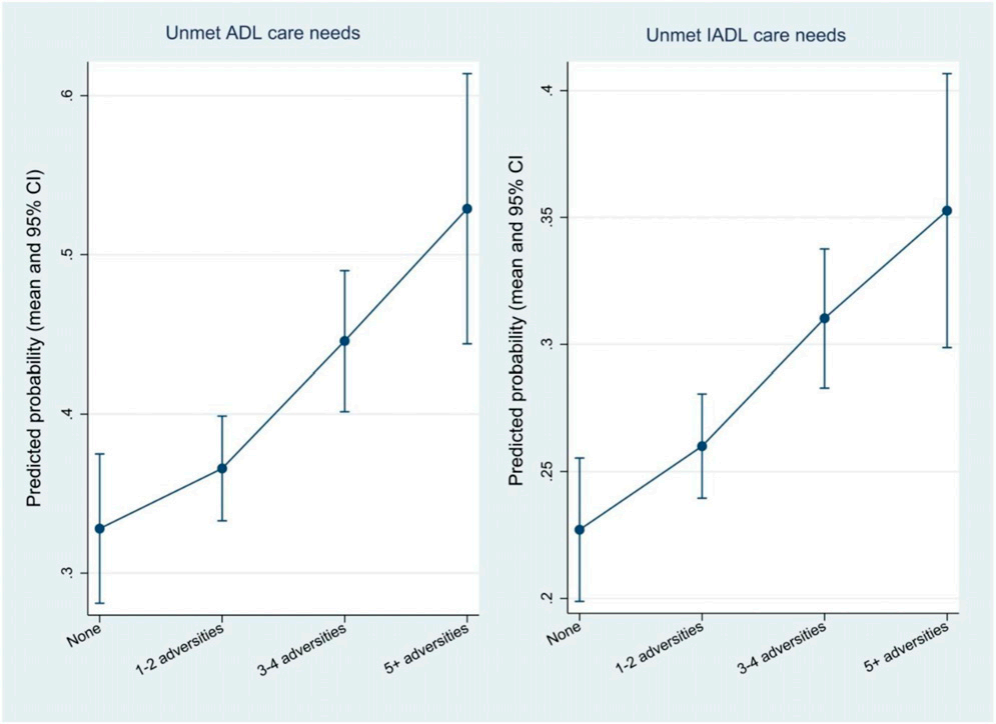
Predicted probability of unmet ADL care needs and unmet IADL care needs according to the number of childhood adversities (mean value and 95% confidence interval).

Table 3 shows the determinants of unmet care needs based on binary logistic regression. The number of childhood adversities was a statistically significant predictor of unmet needs. An increase in one childhood adversity was associated with a 13% increase in the odds of unmet ADL care needs and a 9% increase in the odds of unmet IADL care needs, respectively. For older adults who had not experienced any of the childhood adversities under investigation, the average probabilities of unmet ADL and IADL care needs were 0.33 and 0.23, respectively. For those with more than five adversities, the average probabilities of unmet ADL and IADL care needs increased to 0.53 and 0.35, respectively (Figure 1).

**Table 3.** Association Between Childhood Adversities and Unmet Care Needs (Binary Logistic Regression).

	Unmet ADL care needs	Unmet IADL care needs
	OR (standard error)	OR (standard error)
Number of adversities	1.13** (0.05)	1.09** (0.04)
70–79 years old (60–69 years old)	1.21 (0.21)	1.16 (0.15)
80+ years old (60–69 years old)	1.01 (0.24)	0.60** (0.09)
Female (male)	1.59** (0.26)	0.91 (0.11)
Rural China (urban China)	1.21 (0.21)	1.08 (0.15)
Married people (single people)	0.97 (0.20)	0.60*** (0.09)
Living with others (living alone)	0.89 (0.24)	0.49*** (0.09)
Number of children	0.90* (0.04)	0.97 (0.03)
1 ADL limitation (IADL limitations only)	—	0.87 (0.13)
2+ ADL limitations (IADL/1 ADL limitation)	0.51*** (0.08)	0.47*** (0.08)
Chronic disease	0.98 (0.04)	0.97 (0.03)
Receiving formal education (no education)	1.05 (0.17)	0.95 (0.12)
Receiving pension (no pension)	0.99 (0.16)	0.71** (0.08)

Notes: Categories in the parentheses in column 1 are the reference categories; OR: odds ratio; *P < .05, **P <.01, ***P < .001.

The importance of the control variables varied according to the types of unmet needs. Females and those with fewer children were more likely to have unmet ADL care needs. In contrast, younger people, single people living alone, and those without a pension were more likely to have unmet IADL care needs (Table 3).

Table 4 shows the association between childhood adversities and family relationships and the association between family relationships and unmet needs. We investigated two types of unmet needs (i.e. unmet ADL and IADL care needs, respectively) and two types of family relationships (i.e. relationships with spouse and children, respectively), so there were in total four mediation models. All of the models include the same control variables as in Table 3. For the purpose of readability, we only report the regression results of the key variables. Detailed results for the control variables can be found in the Supplementary appendix. For married older adults with ADL care needs, more childhood adversities were significantly associated with a poorer relationship with their spouse (OR = 1.12, *P*-value = .025), and poorer relationships were significantly associated with a higher probability of unmet needs (OR = 1.27, *P*-value = .028). For married people with IADL care needs, more childhood adversities were also significantly associated with a poorer relationship with their spouse (OR = 1.15, *P*-value < .001), but the association between the relationship with spouse and unmet IADL care needs was not statistically significant (OR = 1.05, *P*-value = .56).

**Table 4.** The Mediating Effects of Family Relationships.

	Ordinal logistic regression	Binary logistic regression
	OR (standard error)	OR (standard error)
Mediation model 1 (*N* = 614)	Relationship with spouse	Unmet ADL care needs
Childhood adversities	1.12* (0.06)	1.13* (0.06)
Relationships with spouse	—	1.27* (0.14)
Mediation model 2 (*N* = 1355)	Relationship with spouse	Unmet IADL care needs
Childhood adversities	1.15*** (0.04)	1.08* (0.04)
Relationships with spouse	—	1.05 (0.08)
Mediation model 3 (*N* = 871)	Relationship with children	Unmet ADL care needs
Childhood adversities	1.12** (0.05)	1.12** (0.05)
Relationships with children	—	1.11 (0.11)
Mediation model 4 (*N* = 1914)	Relationship with children	Unmet IADL care needs
Childhood adversities	1.13*** (0.03)	1.08* (0.04)
Relationships with children	—	1.15* (0.08)

Notes: OR: odds ratio; *P < .05, **P < .01, ***P < .001; imputed dataset with 20 imputations; all control variables are included in each regression model.

For older adults with children and with ADL care needs, those experiencing more childhood adversities were significantly more likely to have poor relationships with their children (OR = 1.12, *P*-value = .018). People with poorer relationships with their children were more likely to have unmet needs, but such an association was not statistically significant (OR = 1.11, *P*-value = .30). For people with children and with IADL care needs, those experiencing more childhood adversities were significantly more likely to have poorer relationships with their children (OR = 1.13, *P*-value < .001), which in turn was significantly associated with unmet needs (OR = 1.15, *P*-value = .042).

Table 5 shows the z-value of the indirect effects calculated using Iacobucci’s (2012) method. Relationship with spouse served as an important mediator of the association between childhood adversities and unmet ADL care needs, whereas the relationship with children was an important mediator of the association between childhood adversities and unmet IADL care needs. The indirect effects derived using the KHB method (Karlson et al., 2012) or from the linear probability models show a similar picture (Table 5). Childhood adversities had a significant indirect effect on unmet ADL care needs through the relationship with spouse and the indirect effects accounted for 17%–18% of the total effect. Childhood adversities had a significant indirect effect on unmet IADL care needs through relationships with children and the indirect effects accounted for 12% of the total effects.

**Table 5.** Indirect effects of childhood adversities on unmet needs through family relationships.

Approach 1: Iacobucci’s (2012) method						
		Relationships with spouse		Relationships with children		
Mediator		Unmet ADL care needs	Unmet IADL care needs	Unmet ADL care needs	Unmet IADL care needs	
Indirect effects		1.84	0.62	0.58	2.30	
*P*-value		0.066	0.537	0.560	0.021	
Approach 2: KHB (2012) method						
Total effects		0.127* (0.057)	0.103* (0.041)	0.136** (0.048)	0.096* (0.034)	
Direct effects		0.104 (0.057)	0.100* (0.041)	0.133** (0.049)	0.084* (0.034)	
Indirect effects		0.023* (0.011)	0.003 (0.006)	0.003 (0.005)	0.012* (0.005)	
% of indirect effects		18%	4%	2%	12%	
Approach 3: Linear probability models						
Total effects		0.030*(0.013)	0.020*(0.008)	0.033**(0.011)	0.020**(0.007)	
Direct effects		0.024 (0.014)	0.019*(0.008)	0.032**(0.011)	0.0177*(0.007)	
Indirect effects		0.006*(0.003)	0.001 (0.001)	0.001 (0.001)	0.0023*(0.001)	
% of indirect effects		17%	5%	3%	12%	

Notes: (Bootstrapped) standard errors in parentheses; *P < .05, **P < .01, ***P < .001.

## Discussion

Drawing on data from a nationally representative sample, this study investigated the relationship between adverse experiences in childhood and unmet long-term care needs in old age in the Chinese context. A growing body of international literature has demonstrated that older adults experiencing multiple childhood adversities are more likely to have poor health and develop long-term care needs (Hempel et al., 2021; Hu, 2021; Hughes et al., 2017; Schafer & Ferraro, 2012). Our analyses show that conditional on the onset of long-term care needs, childhood adversities are a major risk factor for unmet personal care needs and unmet domestic care needs in the older population. The larger the number of adversities experienced in childhood, the higher the probability of unmet needs. Such a finding confirms the first hypothesis of our study.

Previous studies have examined the impacts of living arrangements, marital status, functional limitations and socioeconomic status on unmet needs of older adults (Kennedy, 2001; Lima & Allen, 2001; Liu et al., 2012; Peng et al., 2015). We found that even after we control for those factors, the association between childhood adversities and unmet needs remain statistically significant. Such a finding indicates that early-life experiences may affect care utilisation in old age independent of the contemporary factors. This constitutes a novel contribution of our study to the literature and has important policy implications.

In many countries, the state provides care and support to older adults with lower financial means or restricted access to family caregivers. China has been following other countries’ steps to build such a system (Hu et al., 2020). Those policies can play a meaningful role in reducing care inequality and care deprivation in the older population. However, we argue that simply addressing the contemporary risk factors may not be sufficient because the unmet needs of older adults are the result of cumulative disadvantage throughout the life course and are directly linked to adverse experiences in childhood. Instead, policymakers in the long-term care sector should be more aware of the early life causes of unmet needs. There is a strong case for the government to consider shifting the balance from intervention to prevention programmes.

Most of the childhood adversities we have investigated in this study are closely related to family policy and education policy. A life-course analysis of unmet needs sheds light on the potential benefits of coordination between different policy domains. Policies such as financial support for low-income families with children, providing training in parenting skills, anti-bullying policies and an emphasis on social integration in school are not only important in providing equal opportunity for children and building their social competence, but will also have positive feedback in the future by reducing the risk of unmet care needs.

We have found solid evidence that the quality of family relationships mediates the association between childhood adversities and unmet needs in old age, which confirms the second hypothesis of the study. Older adults experiencing more childhood adversities have a poor relationship with their spouse and children, which is consistent with findings in the existing literature (Ford et al., 2011; Rodgers, 1996; Umberson et al., 2005; Wolke et al., 2013). Most importantly, our study extends the existing knowledge by showing that the association between childhood adversities and poor family relationships may lead to grave consequences in terms of unmet needs and care inequality.

It should be stressed that the mediating effects of family relationships are found to vary according to the sources of care and the type of care needs. The explanation is that spouse and children help with different tasks in the family. The task-specific model posits that people seek help with daily tasks from different caregivers based on the features of those tasks (Messeri et al., 1993). Compared to help with IADL tasks, help with ADL tasks requires a higher level of personal contact, continuous commitment and a shared lifestyle between caregivers and care recipients. According to these criteria, a spouse has a comparative advantage in providing ADL care and is more likely to be the primary care provider, whereas children are more likely to be the primary provider of IADL care. Such a division of labour means that unmet ADL care needs are more sensitive to relationships with spouse, whereas unmet IADL care needs react more strongly to relationships with children.

Despite their importance, relationships with spouse and adult children are unlikely to be the only mediating factors. We estimate that the indirect effects of childhood adversities on unmet needs through the pathway of family relationships account for 12%–18% of the total effects. A large direct effect of childhood adversities remains, and there are at least two possible explanations for this. The first reason concerns older adults’ relationships with other family caregivers such as siblings or grandchildren, which may also be negatively affected by childhood adversities. Some older adults, especially those with severe functional limitations, require long hours of labour-intensive care from a network of caregivers. Good relationships with other relatives help older adults get additional support and ensure that complex care needs can be fully met.

The second one relates to coping strategies. As mentioned previously, childhood adversities compromise the development of attachment security. Apart from poor family relationships, insecure attachment also influences people’s willingness to seek support to cope with care needs. The existing theories posit that there are three forms of insecure attachment: preoccupied style, avoidant/fearful style and dismissive style (Bartholomew, 1990; Mikulincer & Shaver, 2009). Older adults with avoidant or dismissive styles of insecure attachment may have excellent family relationships but prefer to cope with care needs on their own rather than asking for help from their spouse or children because they either fear rejection or harbour serious doubt about the effectiveness of the support.

### Limitations of the Study

Two limitations of this study should be given due attention. First, childhood adversities in the CHARLS survey were reported by older adults retrospectively and thus were subject to recollection bias. Data collected prospectively are most useful to avoid such bias, but cohort studies that follow participants from early childhood to late old age are rare. Second, the causality between the quality of family relationships and unmet needs cannot be assumed. It is possible that unmet needs spoil older adults’ relationships with family members. Longitudinal studies in the future focussing on the reciprocal pathways for these two variables would be useful to disentangle the underlying causal mechanism.

## Conclusion

Unmet long-term care needs represent a major stressor for older adults. The causes of unmet needs are not confined to contemporary factors but can be traced back to a person’s early life and are deeply embedded in the constantly evolving social networks spanning the entire life. Long-term care research based on a life course perspective has great potential to inform the designs of long-term care policies. It can help policymakers and care providers understand better the individual-level variations in care needs, coping strategies and preferred care arrangements that are driven by historical events and past experiences of older adults. This is of great value because long-term care policies across the world are increasingly taking a ‘personalised’ approach in care planning and service delivery. Moreover, childhood adversities and human development are multi-faceted issues, as is the care utilisation of older adults. It is important for the government to take a holistic view and place a greater emphasis on inter-departmental communication and cooperation. Policymakers may want to encourage concerted efforts from multiple policy domains to tackle the unmet care needs of older adults together. Given the scarcity of evidence and the implications for policies, we call for more international research to be conducted in this area. It will be useful to examine whether and how early-life experiences affect the selection of care (e.g. formal vs. informal care) and investigate alternative mediation pathways for the association between childhood adversities and care utilisation.

## Supplemental Material

sj-pdf-1-roa-10.1177_01640275211048237 – Supplemental Material for Childhood Adversities and Unmet Needs of Older Chinese Adults: The Mediation Effects of Family Relationships Click here for additional data file. Supplemental Material, sj-pdf-1-roa-10.1177_01640275211048237 for Childhood Adversities and Unmet Needs of Older Chinese Adults: The Mediation Effects of Family Relationships by Bo Hu and Mingyu Wei in Research on Aging
