# Supplementary material for: Childhood Adversities and Unmet Needs of Older Chinese Adults: The Mediation Effects of Family Relationships
Source: Res Aging. 2021 Oct 13;44(5-6):465–76. doi: 10.1177/01640275211048237 (PMC9039319; doi:10.1177/01640275211048237)
Supplement: sj-pdf-1-roa-10.1177_01640275211048237 – Supplemental Material for Childhood Adversities and Unmet Needs of Older Chinese Adults: The Mediation Effects of Family Relationships [file sj-pdf-1-roa-10.1177_01640275211048237.pdf]

APPENDIX

Table A1 The mediating effects of relationships with spouse, imputed datasets

	**Relationships with spouse**<br>OR (standard error)	**Unmet needs**<br>OR (standard error)
**Older people with ADL care needs (N=614)**		
Childhood adversities	1.12* (0.06)	1.13* (0.06)
Relationships with spouse	-	1.27* (0.14)
70-79 years old (60-69 years old)	1.07 (0.22)	1.22 (0.24)
80+ years old (60-69 years old)	0.94 (0.29)	1.17 (0.36)
Female (male)	1.93*** (0.35)	1.67** (0.31)
Rural china (urban China)	1.11 (0.23)	1.02 (0.22)
Number of children	1.01 (0.06)	0.95 (0.05)
2+ ADL limitations (1 ADL limitation)	1.06 (0.19)	0.62** (0.11)
Chronic diseases	1.04 (0.05)	0.98 (0.04)
Receiving formal education (no education)	1.10 (0.21)	0.96 (0.19)
Receiving pension (no pension)	0.86 (0.16)	1.20 (0.24)
**Older people with IADL care needs (N=1,355)**		
Childhood adversities	1.15*** (0.04)	1.08* (0.04)
Relationships with spouse	-	1.05 (0.09)
70-79 years old (60-69 years old)	1.04 (0.14)	1.22 (0.18)
80+ years old (60-69 years old)	1.09 (0.25)	0.97 (0.25)
Female (male)	2.03*** (0.25)	0.91 (0.13)
Rural china (urban China)	1.16 (0.16)	1.03 (0.17)
Number of children	0.97 (0.04)	0.98 (0.05)
1 ADL limitation (IADL limitations)	0.81 (0.13)	0.79 (0.15)
2+ ADL limitations (IADL limitations)	0.92 (0.15)	0.62** (0.12)
Chronic diseases	1.06* (0.03)	0.97 (0.04)
Receiving formal education (no education)	1.19 (0.15)	0.94 (0.14)
Receiving pension (no pension)	1.04 (0.13)	0.71* (0.1)

Notes: Categories in the parentheses are the reference categories; OR: odds ratio; *p<0.05, **p<0.01, ***p<0.001; imputed dataset with 20 imputations

Table A2 The mediating effects of relationships with children, imputed datasets

	**Relationships with children**	**Unmet needs**
	OR (standard error)	OR (standard error)
**Older people with ADL care needs (N=871)**		
Childhood adversities	1.12** (0.05)	1.12** (0.05)
Relationships with children	-	1.11 (0.11)
70-79 years old (60-69 years old)	0.97 (0.16)	1.18 (0.20)
80+ years old (60-69 years old)	0.83 (0.21)	0.99 (0.23)
Female (male)	0.70* (0.12)	1.70** (0.28)
Rural china (urban China)	0.99 (0.17)	1.17 (0.20)
Married people (single people)	1.06 (0.21)	1.02 (0.21)
Living with others (living alone)	0.74 (0.05)	0.88 (0.23)
Number of children	0.96 (0.05)	0.93 (0.04)
2+ ADL limitations (1 ADL limitation)	0.99 (0.16)	0.49*** (0.07)
Chronic diseases	1.02 (0.04)	0.98 (0.04)
Receiving formal education (no education)	0.84 (0.14)	1.13 (0.19)
Receiving pension (no pension)	0.95 (0.15)	0.99 (0.16)
**Older people with IADL care needs (N=1,914)**		
Childhood adversities	1.13*** (0.03)	1.08* (0.04)
Relationships with children	-	1.15* (0.08)
70-79 years old (60-69 years old)	1.05 (0.13)	1.19 (0.15)
80+ years old (60-69 years old)	0.86 (0.17)	0.65* (0.12)
Female (male)	0.72** (0.08)	0.97 (0.12)
Rural china (urban China)	1.04 (0.13)	1.06 (0.14)
Married people (single people)	1.07 (0.15)	0.65** (0.09)
Living with others (living alone)	0.65* (0.13)	0.58** (0.11)
Number of children		1.01 (0.04)
1 ADL limitation (IADL limitations)	0.93 (0.12)	0.83 (0.12)
2+ ADL limitations (IADL limitations)	0.90 (0.14)	0.51*** (0.08)
Chronic diseases	1.04 (0.03)	0.97 (0.03)
Receiving formal education (no education)	0.93 (0.11)	0.95 (0.12)
Receiving pension (no pension)	0.98 (0.11)	0.73** (0.08)

Notes: Categories in the parentheses are the reference categories; *p<0.05, **p<0.01, ***p<0.001; imputed dataset with 20 imputations

Table A3 The mediating effects of relationships with spouse, completed cases

	**Relationships with spouse** OR (standard error)	**Unmet needs** OR (standard error)
**Older people with ADL care needs (N=472)**		
Childhood adversities	1.13* (0.06)	1.11 (0.07)
Relationships with spouse	-	1.30* (0.14)
70-79 years old (60-69 years old)	1.10 (0.22)	1.47 (0.32)
80+ years old (60-69 years old)	0.92 (0.32)	1.39 (0.51)
Female (male)	1.93*** (0.36)	1.62* (0.34)
Rural china (urban China)	1.08 (0.23)	1.14 (0.27)
Number of children	1.02 (0.06)	0.95 (0.06)
2+ ADL limitations (IADL/1 ADL limitation)	1.08 (0.20)	0.67 (0.14)
Chronic diseases	1.05 (0.05)	1.01 (0.05)
Receiving formal education (no education)	1.08 (0.21)	0.97 (0.21)
Receiving pension (no pension)	0.78 (0.15)	1.27 (0.28)
**Older people with IADL care needs (N=1,102)**		
Childhood adversities	1.16*** (0.04)	1.10* (0.05)
Relationships with spouse	-	1.05 (0.09)
70-79 years old (60-69 years old)	1.05 (0.13)	1.21 (0.19)
80+ years old (60-69 years old)	1.23 (0.31)	0.94 (0.31)
Female (male)	2.00*** (0.25)	0.87 (0.14)
Rural china (urban China)	1.24 (0.17)	1.19 (0.22)
Number of children	0.97 (0.04)	0.95 (0.05)
1 ADL limitation (IADL limitations only)	0.79 (0.12)	0.89 (0.17)
2+ ADL limitations (IADL/1 ADL limitation)	0.92 (0.16)	0.52** (0.13)
Chronic diseases	1.08* (0.03)	1.00 (0.04)
Receiving formal education (no education)	1.25 (0.16)	0.89 (0.14)
Receiving pension (no pension)	1.00 (0.13)	0.73* (0.11)

Notes: Categories in the parentheses are the reference categories; OR: odds ratio; *p<0.05, **p<0.01, ***p<0.001

Table A4 The mediating effects of relationships with children, completed cases

	**Relationships with children**	**Unmet needs**
	OR (standard error)	OR (standard error)
**Older people with ADL care needs (N=626)**		
Childhood adversities	1.12* (0.05)	1.12* (0.06)
Relationships with children	-	1.11 (0.11)
70-79 years old (60-69 years old)	0.99 (0.17)	1.29 (0.25)
80+ years old (60-69 years old)	0.77 (0.21)	1.42 (0.42)
Female (male)	0.70* (0.12)	1.69** (0.32)
Rural china (urban China)	0.97 (0.18)	1.31 (0.27)
Married people (single people)	1.08 (0.24)	0.73 (0.18)
Living with others (living alone)	0.62 (0.19)	1.34 (0.44)
Number of children	0.96 (0.05)	0.96 (0.05)
2+ ADL limitations (IADL/1 ADL limitation)	0.98 (0.16)	0.49*** (0.09)
Chronic diseases	1.03 (0.04)	1.01 (0.05)
Receiving formal education (no education)	0.86 (0.15)	1.07 (0.20)
Receiving pension (no pension)	0.93 (0.16)	0.99 (0.18)
**Older people with IADL care needs (N=1,475)**		
Childhood adversities	1.13*** (0.03)	1.08* (0.04)
Relationships with children	-	1.18* (0.08)
70-79 years old (60-69 years old)	1.05 (0.12)	1.17 (0.16)
80+ years old (60-69 years old)	0.71 (0.14)	0.68 (0.16)
Female (male)	0.73** (0.08)	0.96 (0.13)
Rural china (urban China)	1.01 (0.12)	1.12 (0.17)
Married people (single people)	1.08 (0.16)	0.52*** (0.08)
Living with others (living alone)	0.56** (0.11)	0.71 (0.16)
Number of children	0.94 (0.03)	0.99 (0.04)
1 ADL limitation (IADL limitations only)	0.90 (0.12)	0.93 (0.15)
2+ ADL limitations (IADL/1 ADL limitation)	0.84 (0.13)	0.47*** (0.10)
Chronic diseases	1.06* (0.03)	0.97 (0.03)
Receiving formal education (no education)	0.95 (0.11)	0.97 (0.13)
Receiving pension (no pension)	0.98 (0.11)	0.76* (0.10)

Notes: Categories in the parentheses are the reference categories; *p<0.05, **p<0.01, ***p<0.001
